# Efficacy of relaxin for cisplatin-induced testicular dysfunction and epididymal spermatotoxicity

**DOI:** 10.1186/s12610-020-0101-y

**Published:** 2020-03-09

**Authors:** Tetsuya Kohsaka, Itaru Minagawa, Masashi Morimoto, Takuya Yoshida, Tomohiro Sasanami, Yoshitaka Yoneda, Naoki Ikegaya, Hiroshi Sasada

**Affiliations:** 1 Department of Applied Life Sciences, Animal Reproduction & Physiology Faculty of Agriculture, Shizuoka University, 836 Ohya, Suruga-ku, Shizuoka, 422-8529 Japan; 2 Department of Clinical Nutrition, School of Food and Nutritional Science, University of Shizuoka, Shizuoka, 422-8526 Japan; 3 Advanced Reproductive Medical Center, Shizuoka Ladies Clinic, Shizuoka, 420-0837 Japan; 4 Department of Medicine, Yaizu Municipal General Hospital, Shizuoka, 422-8505 Japan; 5 Division of Animal Science, Kitasato University School of Veterinary Medicine, Towada, 034-8628 Japan

**Keywords:** Cisplatin, Side effect, Testicular dysfunction, Relaxin, Antioxidant effect, Anti-apoptotic effect, Cis platine, effets secondaires, dysfonction testiculaire, relaxine, effet anti-oxydant, effet anti-apoptotique

## Abstract

**Background:**

Cisplatin (CP) is an extremely effective anticancer agent widely used to treat various cancer types, however, the potential side effects include testicular dysfunction. This study was to investigate, using a rat model of CP-induced testicular dysfunction, the protective effects of relaxin (RLN) against oxidative stress, testicular function, histological damage, spermatogenesis, germ-cell apoptosis, and sperm output, and to explore the usefulness of RLN as a potential protective drug for use with CP in chemotherapeutic treatments.

**Methods:**

Sprague-Dawley male rats were used, which were divided into three groups: sham control, CP, and CP + RLN. Porcine RLN (500 ng/h) or saline was infused for 5 days using an implanted osmotic mini-pump following intraperitoneal injection of CP (6 mg/kg). RLN dose was chosen based on previous studies showing that it resulted in serum relaxin levels comparable to those in rats at the middle of pregnancy. At 5 days after CP administration, samples were collected and assessment of testicular histopathology, germ-cell apoptosis, oxidative stress, lipid peroxidation, and sperm quality was performed as main measures.

**Results:**

The testicular CP model showed reduced testis weight and significantly decreased spermatogenesis scores. Additionally, CP administration induced a 4.6-fold increase in the apoptotic index associated with a significant increase in oxidative stress and upregulation of pro-apoptotic *Casp3* and downregulation of anti-apoptotic *Bcl2* levels, resulting in a marked reduction in sperm concentration. However, RLN administration caused a significant reduction in CP-mediated damage by attenuating oxidative stress and cell apoptosis. RLN administration efficiently scavenged ROS via the activation of SOD, CAT, and GPx and upregulation of GSH to prevent lipid peroxidation and decreased apoptosis by altering *Bcl2* and *Casp3* expression, thereby reducing histopathological damage and restoring spermatogenesis. Furthermore, RLN ameliorated attenuated sperm motility in the cauda epididymis resulting from CP treatment.

**Conclusions:**

This study clearly indicates that RLN exerts a protective effect against CP-induced testicular damage through attenuation of oxidative stress and suppression of apoptosis. Our findings suggest RLN as a potentially efficacious drug for use with cisplatin chemotherapy in order to ameliorate CP-induced side effects and testicular injury adversely affecting spermatogenesis, sperm quality, and oxidative-stress parameters.

## Résumé

**Contexte:**

Le cis platine (CP) est un agent anticancéreux extrêmement efficace largement utilisé pour traiter divers types de cancer. Parmi les effets secondaires potentiels associés aux traitements par CP on compte le dysfonctionnement des testicules. Le propos de ce manuscrit est d'étudier, à l'aide d'un modèle rat de dysfonctionnement testiculaire induit par la prise de CP, l’action protectrice de la relaxine (RLN) contre les effets délétères dus au CP lesquels incluent le stress oxydant, la perte de fonction testiculaire, les dommages histologiques au testicule, l'apoptose des cellules germinales et la baisse de la qualité des spermatozoïdes. L’objectif est d’explorer l'utilité de la RLN comme médicament protecteur potentiel à utiliser avec le CP dans les traitements chimiothérapeutiques.

**Méthodes:**

Des rats mâles Sprague-Dawley ont été utilisés. Trois groupes : contrôle, CP, et CP + RLN ont été comparés. Après une injection intrapéritonéale de CP (6mg/kg), de la RLN porcine (500 ng/h) ou du sérum physiologique a été perfusé pendant 5 jours en utilisant une mini-pompe osmotique implantée. La dose de RLN a été choisie en fonction d'études antérieures qui avaient montré qu'elle entraînait des taux sériques de RLN comparables à ceux de rats en milieu de la gestation. Cinq jours après l'administration de la CP, des échantillons ont été prélevés afin d’évaluer l'histopathologie, l'apoptose des cellules germinales, le stress oxydant, la peroxydation des lipides et les paramètres spermatiques.

**Résultats:**

Le groupe CP a montré une réduction du poids des testicules et une diminution significative des scores de spermatogenèse. De plus, l'administration de CP a entraîné une augmentation de l’apoptose de 4,6 fois associée à une augmentation significative du stress oxydant, de la régulation à la hausse de la Caspase 3 pro-apoptotique et à la baisse de Bcl2 anti-apoptotique conduisant *in fine* à une réduction marquée de la concentration en spermatozoïdes. La RLN a ainsi significativement corrigée les effets négatifs du CP en atténuant le stress oxydant et l'apoptose. La RLN a permis d'éliminer efficacement les ROS via l’activation de la triade enzymatique anti-oxydante superoxyde dismutase (SOD)/catalase (CAT)/glutathion peroxydase (GPx) et via la régulation à la hausse du GSH prévenant ainsi la lipopéroxydation. La RLN a par ailleurs diminué les atteintes histopathologiques testiculaires préservant la spermatogenèse. En parallèle, la RLN a amélioré la mobilité spermatique des spermatozoïdes prélevés dans la queue de l’épididyme.

**Conclusions:**

Cette étude montre clairement que la RLN exerce un effet protecteur contre les lésions testiculaires par l'atténuation du stress oxydant et la suppression de l'apoptose induite par le CP. Nos résultats suggèrent que la RLN est un médicament potentiellement pertinent à utiliser afin de diminuer les effets secondaires induits par le CP sur la fonction testiculaire et sur les spermatozoïdes lors de la chimiothérapie cancéreuse.

## Background

Cisplatin (CP) is an extremely effective anticancer agent widely used for the treatment of various types of human cancers, including testicular, ovarian, uterine, lung, and rectal cancers [1]. However, CP treatment exhibits several side effects, such as hepatotoxicity, renal toxicity, and gonadal toxicity [1–3]. For example, in the case of testicular cancer, CP treatment disrupts spermatogenesis [4] and reduces sperm quality [5, 6], leading to temporary or permanent azoospermia [7], so that the clinical use of CP is being restricted. Therefore, the prevention of CP-related side effects is important to enhance the efficacy of anticancer-drug therapy. Although the pathophysiological mechanisms associated with CP-related side effects remain unresolved, oxidative stress, excessive reactive oxygen species (ROS), and apoptosis/necrosis are reported as probable issues [1–3]. Several approaches have been undertaken to reduce or prevent CP-related side effects, including the use of cytoprotective detoxicants such as amifostine and antioxidants such as vitamin E, and functional food materials [2], although some materials appeared to fail to demonstrate any benefit.

Relaxin (RLN), a 6 kDa peptide initially described as a pregnancy-related hormone that is best known for its role in the female reproductive system, has now been identified as a pleiotropic hormone playing multiple and diverse roles in both reproductive and non-reproductive organs [8–10]. In particular, the potential of RLN as a therapeutic agent for renal [11] and heart failure [11–13] has attracted substantial attention, since it has been reported to have antifibrotic, anti-apoptotic, anti-inflammatory, and antioxidant effects in various clinical applications in studies using animal models of acute kidney [14–16] and heart injury [17–19]. Alternatively, RLN has been identified to potentially influence testis function, given that small amounts of RLN appears to be produced in the testis [20, 21] and that its receptor, RLN family peptide receptor 1 (RXFP1; originally called LGR7, leucine-rich G-protein-coupled receptor 7) [22] has been detected therein [21]. Although the precise effect of RLN on the testis remains unclear, there is evidence that it may be involved in the regulation of testosterone production in Leydig cells and in the reduction of germ cell apoptosis; an earlier study indicated that the addition of RLN to the nucleus-free tissue homogenate from adult macaque testes inhibited testosterone production [23]. A subsequent *Rln* knockout study on adult male mice was reported to cause a decrease in sperm formation [24]. Taking these findings together with the antioxidant and anti-apoptotic functions of RLN, it is undoubtedly reasonable to consider that RLN administration could exert its potential effectiveness at attenuating CP-related side effects in testes.

In the present study, we hypothesized that RLN would be therapeutically useful to attenuate CP-related testicular dysfunction. To test the hypothesis, we investigated, using a rat model of CP-induced testicular dysfunction, the protective effects of RLN against oxidative stress, testicular function, histological damage, spermatogenesis, germ-cell apoptosis, and epididymal spermatotoxicity.

## Methods

### Animals and experimental design

Sprague-Dawley male rats (7-weeks old) were purchased from Shizuoka Laboratory Animal Center (Hamamatsu, Japan), housed in a light (14-h light cycle; lights on at 06:00 h) and temperature (23–27 °C)-controlled room, and fed standard food pellets and water ad libitum. All procedures were performed according to the guidelines described in the Shizuoka University of Health Guidelines for the Care and Use of Experimental Animals.

Rats were divided into three groups: sham control (*n* = 6), CP (*n* = 7), and CP + RLN (*n* = 7) groups, because a preliminary study found that control animals treated with RLN were not significantly different from controls not treated with RLN in terms of testicular function, including morphology, spermatogenic status, and apoptosis. CP (cis-Diammineplatinum (II) dichloride; Sigma-Aldrich, St. Louis, MO, USA) was intraperitoneal injected at a single dose (6 mg/kg). The dose of CP was designed according to our pilot study and previous report [16] that demonstrated significant testicular as well as renal damages in rats. RLN or saline infusion started immediately after CP treatment. The RLN used here was purified from pregnant sow ovaries [25]. For continuous infusion of porcine RLN (500 ng/h) or saline for 5 days, animals were anesthetized by intraperitoneal injection of xylazine (Ceractal® 2% solution; Bayer, Osaka, Japan) and pentobarbital sodium (Nembutal® Sodium Solution; Sumitomo Dainippon Pharma, Osaka, Japan) and surgically implanted with an ALZET mini-osmotic pump (Model 2001; DURECT, Cupertino, CA, USA) under the skin. RLN dose was chosen based on a previous study showing that injection of this dose resulted in serum RLN levels comparable to those recorded in mid-pregnant rats [15].

### Sample collection

At 5 days post-CP administration, rats were anesthetized by intraperitoneal injection of xylazine and pentobarbital sodium, and the testes, epididymides, and accessory sex glands were removed and weighed after blood was taken directly from the heart. Blood was centrifuged at 1500 *g* for 15 min at 4 °C, and serum was stored at − 80 °C. One of the testes was rapidly frozen and stored in liquid nitrogen for biochemical analysis, and the other was prepared for histological examination by fixation with 10% neutral-buffered formalin and embedded in paraffin. Epididymal sperm was also collected from cauda epididymides.

### Testosterone assay

Serum testosterone concentrations were measured using a TR-FIA kit (PerkinElmer, Waltham, MA, USA), with an assay detection limit of 0.15 ng/ml and the intra- and inter-assay coefficients of variability of 2.3 and 6.6%, respectively.

### Histological analyses

Testicular specimens were cut at 4-μm sections, stained with hematoxylin and eosin (HE), and examined under a BX50 microscope equipped with a CCD camera (Olympus, Tokyo, Japan) and Instudio V105 software (Pixera, Kanagawa, Japan). The diameters of seminiferous tubules were morphometrically determined using transverse sections of each seminiferous tubule from the experimental groups according to a previously described method [26]. Johnsen’s score was used to categorize spermatogenesis [27]. Each tubular section was given a score ranging from 10 to one according to the presence or absence of the main cell types arranged in the order of maturity. Furthermore, the histopathological parameters by Cosentino et al. [28], including degeneration of the germ cell layer, disarray of germ cell layers, loss of spermatozoa/spermatids, arrested germ cells in different stages of division, and necrotic germ cells, were used to assess overall histological damage, with each testis scored zero to four.

### Quantitative real-time RT-PCR (qPCR)

Total RNA from testes was extracted using ISOGEN reagent (Nippon Gene, Tokyo, Japan), treated with RNase-free DNase I (Qiagen, Hilden, Germany), and reverse transcribed using an oligo(dT)18 primer with ReverTra Ace (Toyobo, Tokyo, Japan). Quantification of complementary DNA (cDNA) from each testis was measured using Bio Photometer (Eppendorf, Hamburg, Germany), followed by dilution with nuclease-free water. Aliquots of diluted cDNA were stored at − 20 °C.

Levels of target gene expression were determined using 100 ng of template cDNA per reaction, and quantified on either a Prism 7000 Real Time PCR System (Life Technologies, Carlsbad, CA, USA) or a Thermal Cycler Dice Real-Time System III TP950 (Takara Bio, Shiga, Japan) with appropriate probes or primers. The expression of each gene was normalized to *glyceraldehyde 3-phosphate dehydrogenase (Gapdh)* levels and quantified by using the 2^−ΔΔCq^ method [29].

### Apoptosis analyses

Apoptotic germ cells in deparaffinized sections were detected by terminal deoxynucleotidyl transferase-mediated dUTP nick end-la beling (TUNEL) assay using an ApoTag peroxidase in situ apoptosis detection kit (EMD Millipore, Temecula, CA, USA). The number of TUNEL-positive cells was counted in each seminiferous tubule. An apoptotic index was calculated by counting the number of round seminiferous tubules expressing greater than three TUNEL-positive cells, dividing by the total number of essentially round seminiferous tubule, which ranged from 102 to 182 in each animal, and multiplying the product by 100, as described previously [30]. Additionally, the expression of the proapoptotic *caspase 3* (*Casp3*) and the antiapoptotic *B-cell lymphoma 2 (Bcl2)*, as well as that of *Rxfp1* and the pro-inflammatory cytokine *interleukin 6 (Il6)*, was detected by qPCR. Cycling conditions were as follows: 95 °C for 2 min, and 45 cycles of 15 s at 95 °C and 1 min at 60 °C; reactions were performed in triplicate on a Prism 7000 Real Time PCR System (Life Technologies). TaqMan Fast Advanced Master Mix (Life Technologies) was used in a final volume of 20 μl containing *Casp3*, *Bcl2*, and *Gapdh* probes designated Rn00563902_m1, Rn99999125_m1, and Rn99999916_s1, respectively (TaqMan Gene Expression Assay; Life Technologies). PowerSYBR Green PCR Master Mix (Life Technologies) was used in the same volume and conditions with primers specific for *Il6*, and *Rxfp1* (Additional file 1: Table S1).

### Antioxidant system

#### qPCR analyses

The expression of genes encoding the antioxidant enzymes *superoxide dismutase 1* (*Sod1*), *catalase* (*Cat*), and *glutathione (GSH) peroxidase 1* (*Gpx1*), as well as enzymes involved in GSH synthesis, namely *GSH reductase (Gsr)* and *GSH synthase (Gss)*, was detected by qPCR. Additionally, we examined gene expression of *Cyp11a1* encoding the rate-limiting enzyme P450scc for steroid biosynthesis. Cycling conditions were as follows: 95 °C for 2 min, and 40 cycles of 5 s at 95 °C and 30 s at 60 °C. Reactions were performed in triplicate on a Thermal Cycler Dice Real-Time System III TP950 (Takara Bio). TaqMan Fast Advanced Master Mix (Life Technologies) was used in a final volume of 20 μl containing *Sod1, Cat, Gpx1, Gsr, Gss, Cyp11a1* and *Gapdh* probes designated as Rn00566938_m1, Rn00560930_m1, Rn00577994_g1, Rn01482159_m1, Rn00564188_m1, Rn00568733_m1, and Rn99999916_s1, respectively (TaqMan Gene Expression Assay; Life Technologies).

#### Biochemical analyses

Testicular samples were homogenized in ice-cold 5 mM phosphate buffer (PB; pH 7.0) with an ULTRA-TURRAX T8 (IKA, Staufen, Germany), and centrifuged at 10,000 *g* for 20 min at 4 °C, after which the supernatant was subjected to the following assays. Protein concentrations were determined by the method of Lowry-Folin [31] with BSA as a standard.

Superoxide dismutase (SOD) was measured using a commercially available assay kit (Dojindo Laboratories, Kumamoto, Japan). The SOD assay was based on the generation of superoxide radicals produced by the xanthine–xanthine oxidase system, which reacts with nitroblue tetrazolium (NBT) to form a red formazan dye. SOD activity is then measured at 450 nm to determine the degree of inhibition of this reaction. One unit of SOD was defined as the enzyme activity causing 50% inhibition of the NBT reduction rate. SOD-like activity was expressed as U/mg protein.

CAT was measured according to the method of Johansson and Borg [32] and based on the reaction of CAT with methanol in the presence of a known concentration of H_2_O_2_. The formaldehyde produced was measured at 510 nm using Purpald solution (Sigma-Aldrich) as a chromogen. CAT-like activity was expressed as nmol/min/mg protein.

GPx was assayed according to the method of Yazdanparast et al. [33], with the activity measured based on the principle that oxidized GSH produced by GPx is reduced at a constant rate by GSH reductase using NADPH (Wako Pure Chemicals, Osaka, Japan) as a cofactor, which allows maintenance of predictable levels of reduced GSH. The oxidative rate of NADPH was monitored at 340 nm, and GPx activity was measured as nmol NADPH/min/mg protein.

Antioxidant GSH level was estimated according to the method of Ellman [34], which is not sensitive. Absorbance was noted spectrophotometrically at 412 nm, and GSH concentration was expressed as μmol/mg protein.

The lipid peroxidation marker and indirect indicator of ROS, malondialdehyde (MDA) was measured using a commercially available assay kit (BIOXYTECH MDA-586; OxisResearch, Portland, OR, USA), and MDA concentration was measured as nmol/mg protein.

### Analyses of sperm morphology, movement and MDA levels

Epididymal sperm was collected from cauda epididymides as described by others [35]. Briefly, the cauda epididymis was placed in modified Krebs−Ringer bicarbonate medium (m-KRB; pH 7.8) [36], minced with scissors to release spermatozoa and incubated for 30 min at 37 °C in an atmosphere of 5% CO_2_, after which the spermatozoa were used to evaluate sperm count, quality, motility and MDA levels.

It should be noted that sperm count results are relative, given that collection of sperm from different males can give significantly different results. The quality of epididymal sperm was assessed based on sperm morphology, as described previously [37]. To assess sperm morphology, the percentages of dead, normal sperm, as well as abnormalities in the sperm head and tail were estimated by eosin-nigrosine staining [38, 39]. Sperm movement was analyzed using a Cellsoft CASA (Cryo Resources, Montgomery, NY, USA) [37, 38]. Sperm samples were diluted to 8 × 10^6^/ml with m-KRB, introduced into the 2X-CEL chamber (Hamilton Thorne, Beverly, MA, USA) on a 37 °C heated microscope stage (Microwarm Plate 30; Kitazato, Tokyo, Japan), and videotaped multiple viewing areas. The percentage of motile sperm, curvilinear velocity (μm/s), linearity, beat-cross frequency (Hz), the mean amplitude of lateral head distance (ALH; μm) and the percentage of circular cells were determined for at least 1000 spermatozoa in each sample. For MDA measurement, spermatozoa were extracted by sonication in ice-cold 5 mM PB, and centrifuged at 10,000 *g* for 5 min at 4 °C, after which the supernatant was subjected to MDA assay.

### Statistical analysis

Values were presented as means ± S.E.M. Data were analyzed by one-way ANOVA, together with Fisher’s Protected Least Significant Difference multiple range test using JMP 10 software (SAS Institute, Cary, NC, USA) to compare the means of different groups. *P* < 0.05 was considered statistically significant.

## Results

### RLN improves testicular function after CP treatment

Although there is no difference in testis weight among groups, diameter of the seminiferous tubule, and serum testosterone level (Table 1) were significantly decreased in the CP group. Additionally, epididymis and accessory gland weights were adversely affected by CP treatment (Table 1). However, RLN administration caused a significant increase in seminiferous tube diameter, and testosterone concentration (Table 1), all of which represent parameters of testicular function.

**Table 1:** Reproductive-organ weights, seminiferous- tube diameter, and serum testosterone levels in all groups

Parameters	Control	CP	CP + RLN
Body weight (g)	266.7 ± 3.0^a^	209.6 ± 3.0^b^	225.6 ± 4.6^c^
Testis (mg)	1465.3 ± 40.3^a^	1366.6 ± 34.1^a^	1461.7 ± 32.4^a^
Seminiferous tubular diameter (μm)	272.7 ± 3.2^a^	240.1 ± 6.7^b^	265.7 ± 4.1^a^
Testosterone levels (ng/m1)	7.1 ± 1.5^a^	1.0 ± 0.8^b^	9.4 ± 2.6^a^
Epididymis (mg)	296.3 ± 13.1^a^	236.7 ± 14.6^b^	282.3 ± 8.8^a^
Prostate gland (mg)	210.2 ± 38.4^a^	104.6 ± 15.8^b^	116.1 ± 9.9^b^
Seminal vesicle (mg)	628.0 ± 74.4^a^	261.6 ± 37.9^b^	366.4 ± 50.0^b^

Values are the means ± S.E.M. and values with different letters are significantly different (*P <* 0.05)

### RLN improves CP-mediated histological damage

**Fig. 1:**
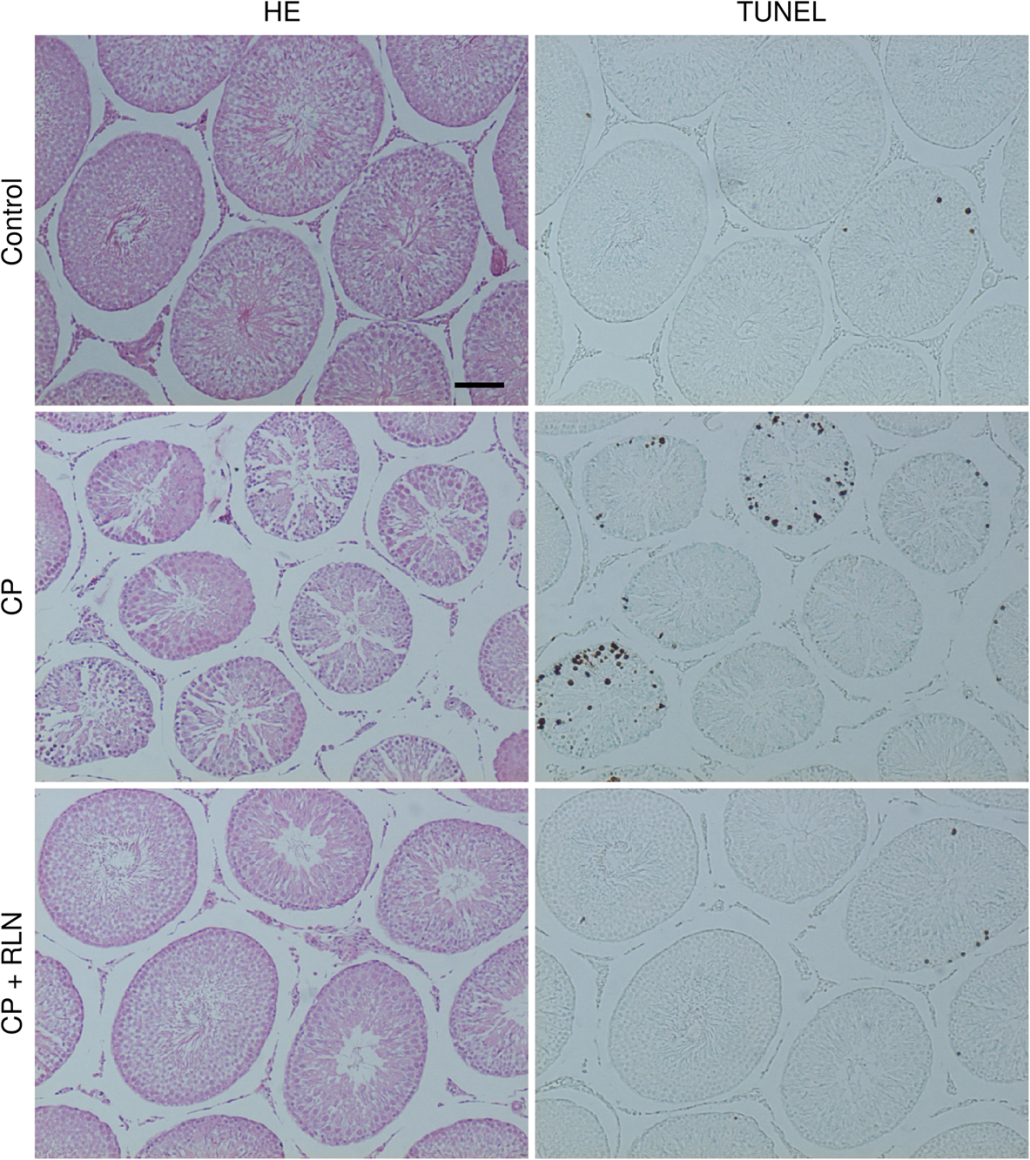
HE staining and TUNEL assay of cross-sections of testis tissue from rats treated with Control (vehicle), CP, and CP + RLN. CP treatment disrupted the seminiferous epithelium. Specifically, seminiferous germ cells were often missing, and spermatogenesis was partially disorganized. Apoptosis was prominent following CP treatment and mainly observed in spermatogonia and spermatocytes existing around the seminiferous tubule. Bar = 100 μm; all panels are the same magnification

**Fig. 2:**
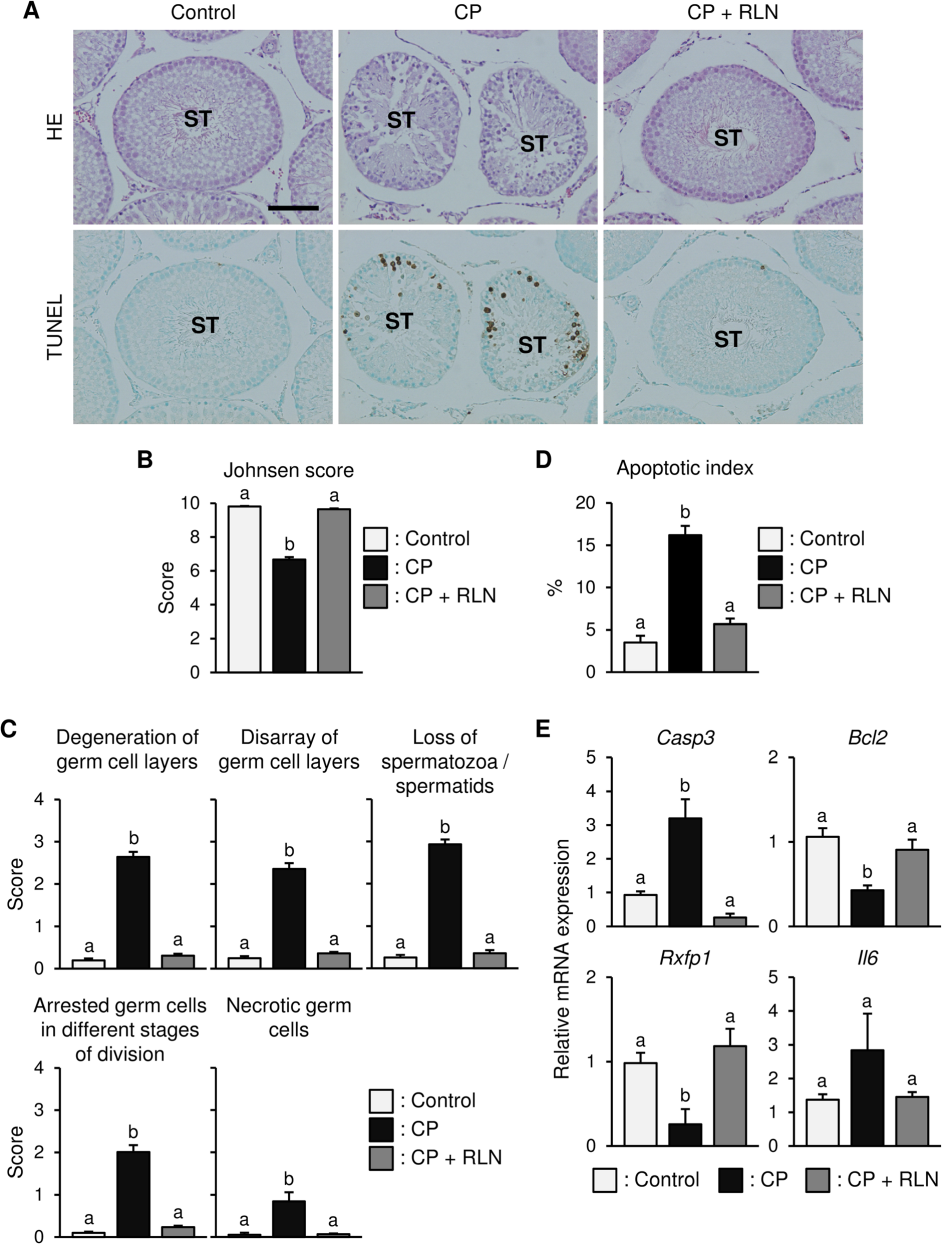
Effects of RLN on testicular damage, and germ cell apoptosis in CP-treated rats. **a** HE staining and TUNEL analysis of rat testis tissue specimens. CP disrupted seminiferous germ cells in the testis in contrast to RLN administration or controls, which both displayed normal testicular tissue structure. Apoptosis was observed mainly in spermatogonia and spermatocytes. ST, seminiferous tubules. Bar = 100 μm; all panels are the same magnification. **b** Johnsen’s testicular spermatogenic score. The Johnsen score showed a significantly lower value following CP treatment, but significantly elevated after RLN administration. **c** Histological damage scores by Cosentino et al. [39]. CP group showed degenerative changes, including degeneration of the germ cell layer, disarray of germ cell layers, loss of spermatozoa/spermatids, arrested germ cells in different stages of division, and necrotic germ cells. These indications of damages were significantly improved following RLN administration. **d** Percentages of seminiferous tubules expressing greater than three TUNEL-positive cells (Apoptotic index, %). **e** qPCR analysis of apoptosis-related gene expression. *Casp3* levels were upregulated, and *Bcl2* levels were downregulated following CP treatment as compared with controls, whereas RLN administration significantly decreased and increased *Casp3* and *Bcl2* expression, respectively. *Rxfp1* levels were downregulated following CP treatment but significantly upregulated after RLN administration, whereas *Il6* expression was unchanged among the three groups. Values represent the means ± S.E.M.; values with different letters are significantly differences (*P* < 0.05)

CP treatment disrupted the seminiferous epithelium (Fig. 1 and 2a). Specially, seminiferous germ cells were often missing, and spermatogenesis was partially disorganized. The Johnsen score [27], which is a widely used quantitative histological grading system for categorizing spermatogenesis, showed a significantly lower value following CP treatment (Fig. 2b). In addition, quantitative assessment of testicular damage by Cosentino et al. [28] indicated that the testes in the CP group showed degenerative changes, including degeneration of the germ cell layer, disarray of germ cell layers, loss of spermatozoa/spermatids, arrested germ cells in different stages of division, and necrotic germ cells (Fig. 2c). These indications of damages were significantly improved following RLN administration (Fig. 2c).

### RLN reduces the proportion of CP-induced germ cell apoptosis

TUNEL analysis revealed apoptosis in the spermatogonia and spermatocyte, with occasional staining in only a few round and elongated spermatids (Fig. 1 and 2a). CP treatment significantly induced a 4.6-fold increase in the apoptotic index relative to controls (Fig. 2d). However, RLN administration led to a significant reduction in the number of apoptotic cells relative to that observed in the CP group (Fig. 2a and d). Moreover, qPCR analysis revealed upregulation of pro-apoptotic *Casp3* and downregulation of anti-apoptotic *Bcl2,* as well as *Rxfp1* (*P* < 0.05), in CP-treated animals as compared with controls (Fig. 2e). However, RLN administration caused a significant decrease in *Casp3* expression and a significant increase in *Bcl2* and *Rxfp1* expression (Fig. 2e)*.* By contrast, *Il6* expression in the testes tended to be higher in CP-treated animals, and RLN administration did not change these levels (Fig. 2e).

### RLN attenuates oxidative stress associated with CP-related injury

**Fig. 3:**
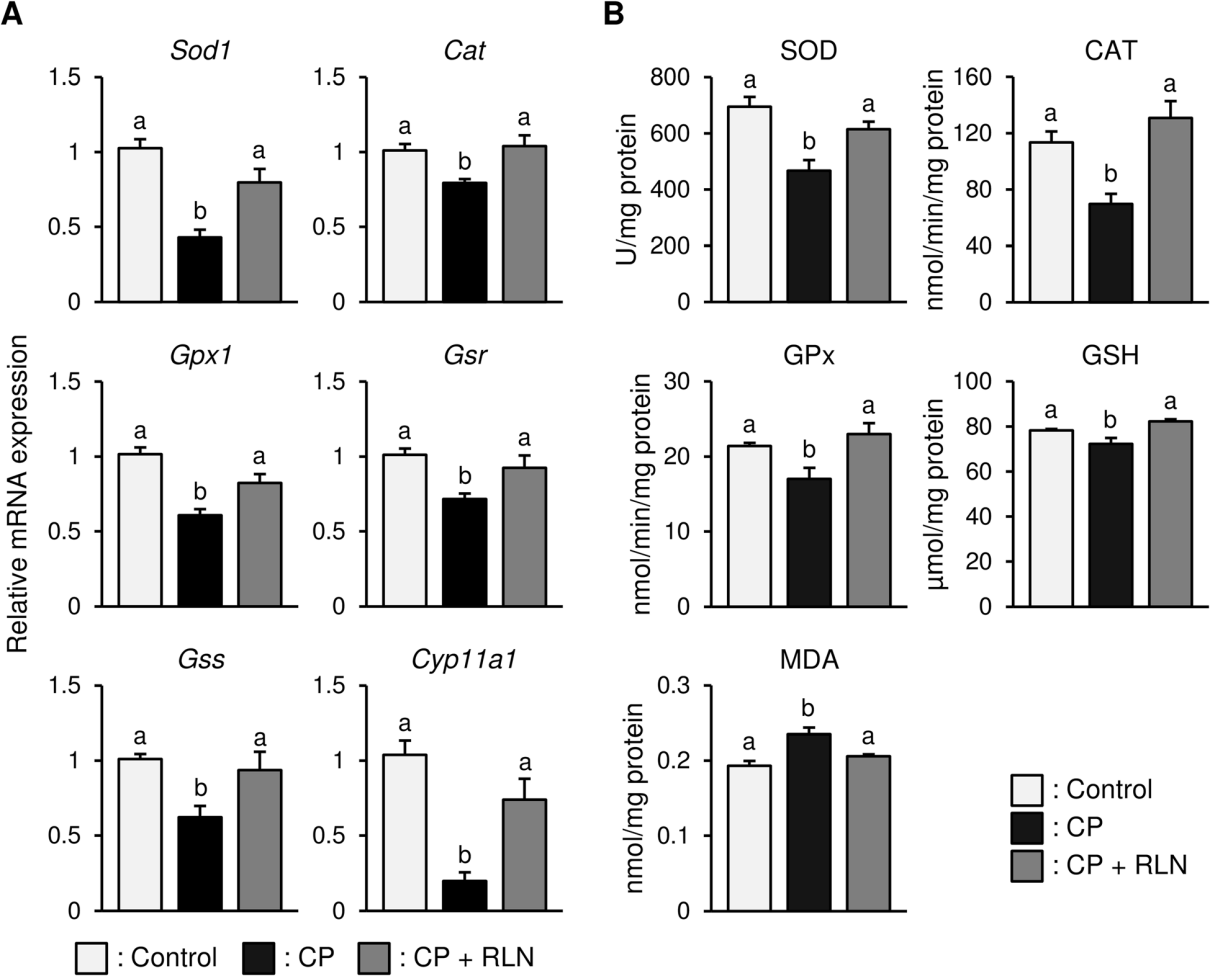
Effects of RLN on oxidative stress in the testes of CP-treated rats. **a** qPCR analysis. CP treatment significantly (*P* < 0.05) downregulated *Sod1, Cat, and Gpx1* expression, as well as that of *Gsr* and *Gss*, all of which were subsequently significantly (*P* < 0.05) upregulated following RLN treatment. The expression of *Cyp11a1* followed a similar pattern. **b** Testicular SOD, CAT, and GPx activities and GSH levels significantly decreased following CP treatment as compared with the control group; however, RLN administration restored these activities/levels. By contrast, testicular MDA levels significantly increased following CP treatment and significantly decreased after RLN administration. Values represent the means ± S.E.M.; values with different letters are significantly differences (*P* < 0.05)

qPCR analysis revealed a significant downregulation (*P* < 0.05) of genes encoding antioxidant enzymes *Sod1, Cat, and Gpx1*, as well as the GSH-synthesis-related enzymes *Gsr* and *Gss*, in CP-treated animals relative to controls (Fig. 3a). However, RLN administration caused a significant upregulation (*P* < 0.05) in *Sod1, Cat,* and *Gpx1* expression, as well as that of *Gsr and Gss* (Fig. 3a). Additionally, CP treatment significantly (*P* < 0.05) downregulated *Cyp11a1* (P450scc) expression, which was subsequently significantly (*P* < 0.05) upregulated following RLN treatment (Fig. 3a).

By contrast, biochemical analysis revealed that testicular SOD, CAT and GPx activities, as well as GSH level, were significantly decreased in the CP group relative to the control group; however, RLN administration significantly increased SOD, CAT and GPx activities, as well as GSH level, in the CP + RLN group (Fig. 3b). By contrast, tissue MDA levels significantly increased following CP-related testicular injury, with a significant decrease in these levels observed following RLN administration (Fig. 3b).

### RLN ameliorates attenuated sperm quality and output in the cauda epididymis resulting from CP treatment

**Fig. 4:**
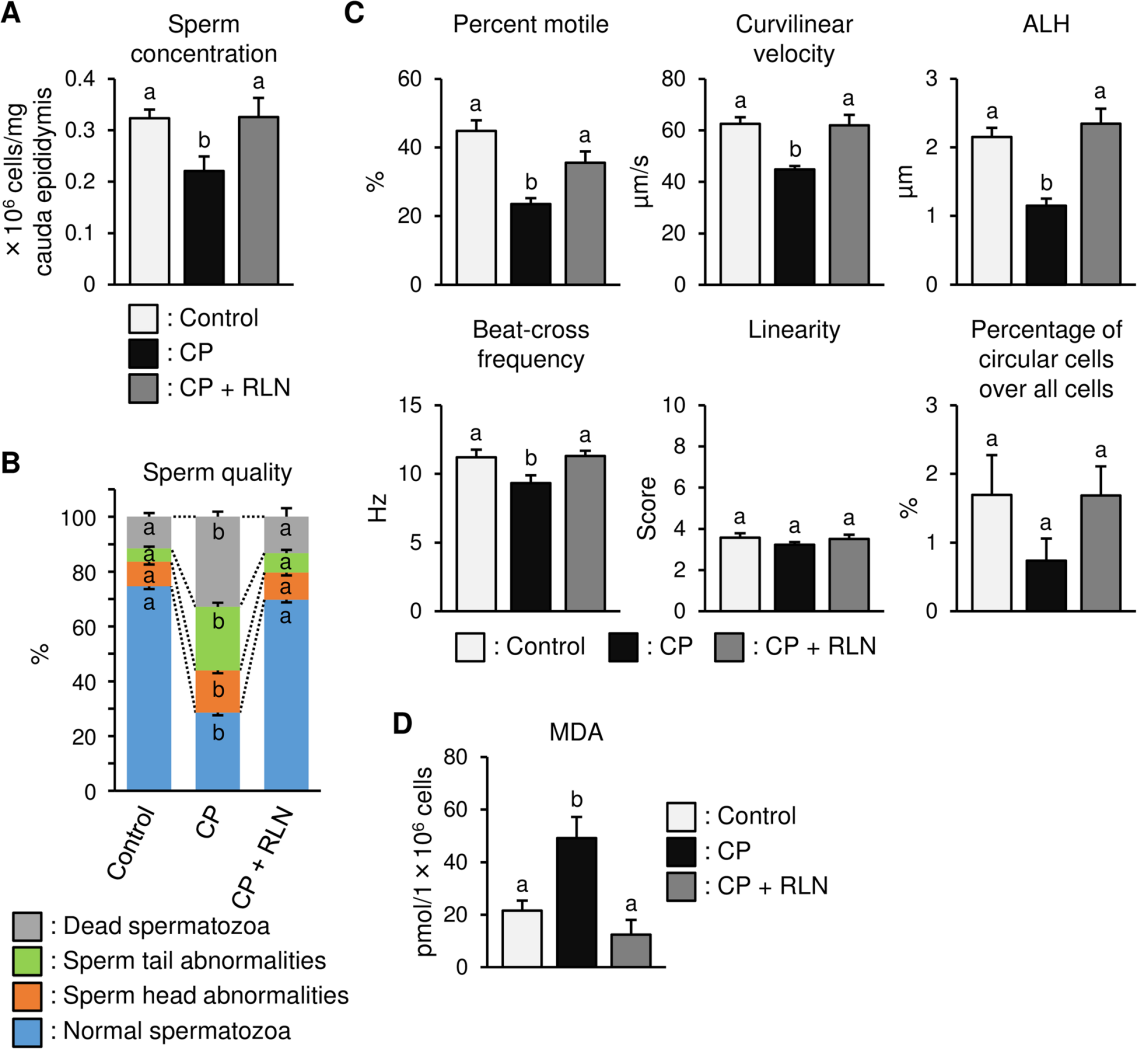
Effects of RLN on sperm count, quality, motility and MDA levels in CP-treated rats. **a** Sperm count per mg cauda epididymis. CP treatment significantly reduced sperm concentration, which significantly increased after RLN treatment. **b** Sperm quality assessed based on morphology. CP treatment reduced the percentage of normal spermatozoa and substantially increased that of dead and abnormal spermatozoa. RLN administration significantly recovered sperm quality to control levels. **c** Sperm-movement characteristics. RLN administration to CP-treated rats significantly prevented CP-induced side effects in motility characteristics, including percent motile, velocity, ALH, and beat-cross frequency. **d** Sperm MDA levels. The sperm MDA levels significantly increased following CP treatment and significantly decreased after RLN administration. Values represent the means ± S.E.M.; values with different letters are significantly differences (*P* < 0.05)

Epididymal sperm concentration significantly decreased in the CP-treated group as compared with that in the control group (Fig. 4a). However, this result is relative, because collection of sperm from different males can give significantly different results. Furthermore, a short period of waiting (5 days) to collect spermatozoa is insufficient to reduce the number of spermatozoa in the epididymis or testis. Therefore, the sperm count obtained here appears to reflect motility rather than problems in the production of spermatozoa. We observed a difference in sperm quality according to morphological assessment, and CP treatment reduced the percentage of normal spermatozoa and substantially increased that of dead and abnormal spermatozoa (Fig. 4b). In abnormal spermatozoa, head abnormalities were observed as irregularly shaped head, such as lacking the usual hook, whereas tail abnormalities involved bent or coiled morphologies. However, RLN administration promoted significant recovery of sperm concentration (Fig. 4a) and quality (Fig. 4b) to levels similar to those observed in the control group. Moreover, sperm movement analysis revealed that RLN administration to CP-treated rats significantly prevented CP-induced side effects in sperm movement characteristics, including percent motile, velocity, ALH, and beat-cross frequency but not including linearity or the percentage of circular cells (Fig. 4c). Furthermore, CP treatment significantly increased MDA levels, which were subsequently significantly attenuated following RLN administration (Fig. 4d).

## Discussion

This is the first study demonstrating the beneficial effects of RLN in CP-induced testicular injury, which represents the CP-related-side effects of gonadal toxicity. The present study demonstrated that CP treatment increased germ-cell apoptosis and histological damage and resulted in disorganized spermatogenesis accompanied by a significant increase in oxidative stress. However, RLN administration significantly reduced the adverse consequences associated with CP-related injury by attenuating oxidative stress and inhibiting apoptosis.

CP alkylates DNA via guanine bases in order to form intra-strand DNA crosslinks, which interfere with DNA-repair mechanisms, thereby inducing apoptosis by activation of p53 and cell cycle arrest [1, 40]. Recently, CP was reported to generate excess amounts of ROS, thereby depleting activity by the antioxidant system, increasing lipid peroxidation, denaturing structural proteins, and promoting cell apoptosis [1, 40]. Specifically, CP results in an increased production of superoxide anion radicals (O_2_•^−^) and promoting hydrogen peroxide (H_2_O_2_) formation, which can be transformed into hydroxyl free radicals (•OH). Hydroxyl free radicals are extremely reactive and can react with polyunsaturated fatty acids in membranes to form an extremely toxic aldehyde. SOD is the first defensive step that detoxifies superoxide anion to produce H_2_O_2_, which is then converted to H_2_O by CAT and GPx. GSH is capable of preventing damage to important cellular components caused by ROS, such as free radicals (O_2_•^−^ and •OH), H_2_O_2_, and toxic lipid peroxides. GSH and these antioxidant enzymes are activated to minimize ROS overload; however, depletion of the scavenging systems results in elevated ROS in the tissues, which promotes Ca^2+^ influx into cells and activation of caspase-dependent or -independent apoptosis pathways [1, 40]. In the present study, CP treatment resulted in testicular dysfunction characterized by lower testicular weight, mean seminiferous tubular diameter, serum testosterone level, and Johnsen’s testicular spermatogenic score [27] and elevated histological damage scores by Cosentino et al. [28] and germ-cell apoptosis. Additionally, we observed significant decreases in the activities of the antioxidant enzymes SOD, CAT, and GPx along with significantly decreased levels of the antioxidant GSH following CP treatment and accompanied by significant increases in MDA levels and germ-cell apoptosis. MDA is generated by ROS and represents a stable terminal product of lipid peroxidation. As such, MDA is used as an indirect indicator of ROS based on the high reactivity and short half-life of ROS making it difficult to quantify [41, 42]. Our findings were substantially consistent with previous reports showing that CP treatment causes adverse effects in the testis by inducing oxidative stress and apoptosis [43–49].

**Fig. 5:**
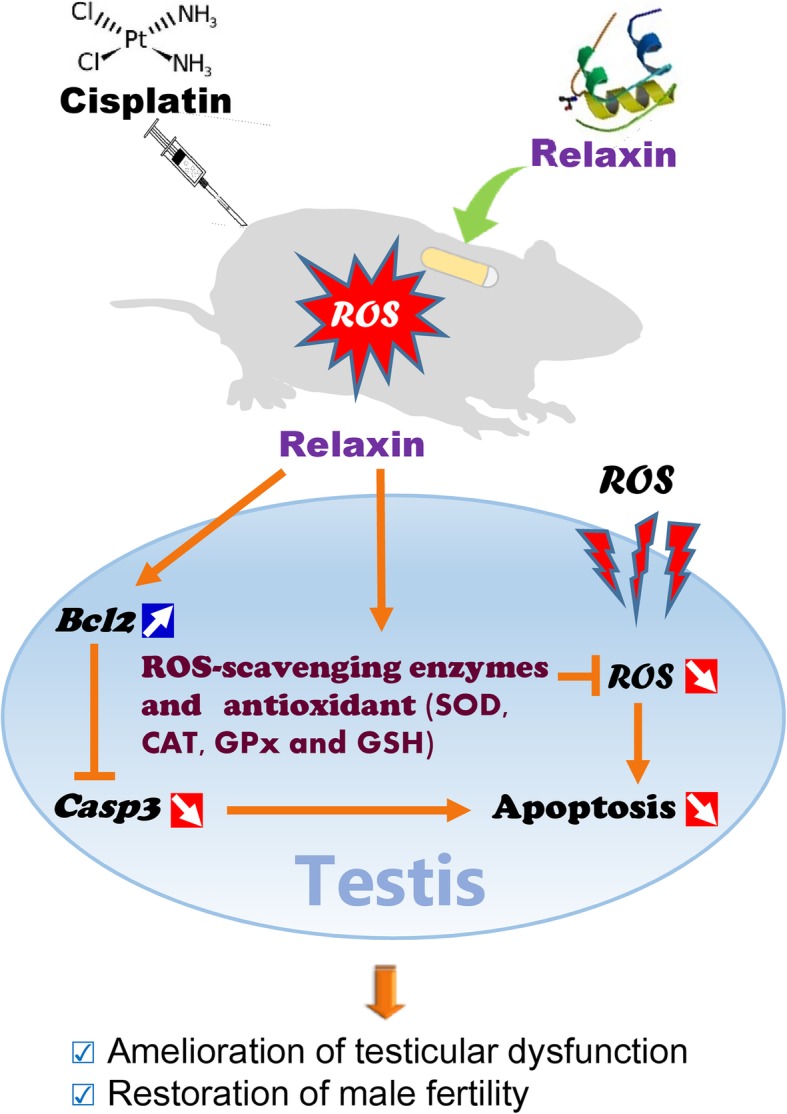
Schematic demonstration of efficacy of relaxin to cisplatin-induced testicular dysfunction. RLN administration efficiently scavenged ROS via activation of SOD, CAT, and GPx and upregulation of GSH to prevent lipid peroxidation and decreased apoptosis by altering *Bcl2* and *Casp3* expression, thereby reducing histopathological damage and restoring spermatogenesis

The present study demonstrated for the first time that RLN ameliorated CP-induced testicular damage by suppressing oxidative stress and apoptosis in adult male rats. Our results clearly showed that RLN administration upregulated the expression of genes encoding the antioxidant enzymes *Sod1*, *Cat*, and *Gpx1*, and GSH-synthesis-related enzymes *Gsr* and *Gss*, thereby activating these antioxidant enzymes and increasing GSH levels to prevent lipid peroxidation. This in turn promoted efficient ROS scavenging and decreased apoptosis by altering *Bcl2* and *Casp3* expression, thereby reducing histopathological damage and restoring spermatogenesis (Fig. 5). Previous studies have reported the antioxidant effects of RLN in myocardial ischemia/reperfusion-induced damage by showing a significant reduction of MDA levels following RLN administration in animal models [50, 51]; however, the mechanisms associated with RLN-mediated suppression of oxidative stress remain unclear. Endogenous RLN appears to be at extremely low levels in the rat testis based on the difficulty of antibody detection [20], which as confirmed by detection of weak *Rln* expression according to RT-PCR in a preliminary study. Additionally, it is possible that endogenous RLN does not play a pivotal role in the testis, as suggested by study of *Rxfp1*-deficient male mice [52]; however, another study reported impaired spermatogenesis due to increased germ-cell apoptosis in *Rln*-knockout mice [24]. Interestingly, in the present study, we found that RLN administration to CP-treated rats upregulated *Rxfp1* expression. Therefore, the results of the present study suggest a possible explanation of how RLN-mediated *Rxfp1* upregulation activates receptor signaling, thereby inducing the expression of genes encoding antioxidant enzymes and those involved in GSH synthesis, increasing antioxidant enzyme activity and GSH level, and contributing to decreases in oxidative stress via inhibiting ROS production or neutralizing ROS [51].

We found a CP-mediated decrease in peripheral blood testosterone concentration, likely resulting in a decrease in testosterone concentration in testes, which is fundamentally consistent with previous reports [43, 44, 53]. It is now accepted that Leydig cells produce testosterone in response to LH and that testosterone acts as a paracrine factor that diffuses into the seminiferous tubules and is required for maintaining spermatogenesis [54]. Although little is known concerning the mechanisms involved in the cytotoxic effects associated with CP on testosterone secretion, CP reportedly strongly inhibits testosterone production through a mechanism involving ROS-mediated P450scc inhibition in Leydig cells [53]. In fact, we found that CP treatment significantly downregulate *Cyp11a1* (P450scc) expression, which was subsequently significantly upregulated following RLN administration. Therefore, the amelioration in serum testosterone level observed in RLN administration to CP-treated rats is likely due to the upregulation of P450scc levels by suppression of ROS in Leydig cells.

In addition to the testis, we found that CP treatment caused spermatotoxicity at the level of the epididymis. CP-induced spermatotoxicity was confirmed by increased MDA and elevated number of dead sperms and morphologically abnormal sperm, as well as a depleted sperm count, due to drug cytotoxicity and diminished sperm motility, which is fundamentally consistent with previous findings [6, 45, 46]. Spermatozoa are especially susceptible to peroxidative damage due to high concentrations of polyunsaturated fatty acids and low antioxidant capacity [55, 56]. We determined that elevations in sperm MDA levels following CP treatment were due to an increase in lipid peroxidation, which destroyed the lipid matrix structure in the membranes of spermatozoa and caused loss of sperm motility, presumably through decreased sperm viability, defective membrane integrity, and increased morphologic defects [6].

It is important to note how RLN improved sperm quality in the treated rats. The present study provides the first evidence that RLN administration to CP-treated rats resulted in a significant decrease in sperm MDA, along with significantly recovery of sperm quality to control levels. The associated decrease in lipid peroxidation in spermatozoa suggested that RLN potently scavenged free radicals (O_2_•^−^ and •OH), resulting in improved sperm quality.

Similar to RLN, melatonin mitigates the deleterious side effects of CP by exerting antioxidant and anti-apoptotic effects in testes, with increased sperm count and motility and decreased abnormal forms [57]. Additionally, dietary antioxidants, such as lycopene [45], Roselle and Ginger [47], royal jelly [48], and rutin [49], as well as plant [44] or fruit extracts [46], have been reported to exert protective effects against CP-induced testicular toxicity. Therefore, RLN might represent a potentially effective drug option for use in cisplatin chemotherapy.

## Conclusions

In conclusion, this study clearly indicates that RLN exerts a protective effect against CP-induced testicular damage through attenuation of oxidative stress and suppression of apoptosis. Our findings suggest RLN as a potentially efficacious drug for use with CP in chemotherapeutic treatments in order to ameliorate CP-induced side effects and testicular injury adversely affecting spermatogenesis, sperm quality, and oxidative-stress parameters. However, this conclusion has an important limitation, because the CP treatment used in this study represented a single dose of chemotherapeutic agent. Usually, CP treatment used in humans is combined with other chemotherapeutic agents, such as bleomycin, etoposide, or corticosteroids. Therefore, further investigation is needed to confirm translation of these results into therapeutics applicable to humans undergoing cancer treatment.

## Supplementary information

**Additional file 1.** List of primers employed for quantitative real- time RT- PCR in rat target genes.

## Data Availability

All data generated or analysed during this study are included in this published article [and its supplementary information files].

## Abbreviations

ALH: Mean amplitude of lateral head distance
Bcl2: B-cell lymphoma 2
CASA: Automated computer-assisted sperm motility analyser
Casp3: Caspase 3
CAT: Catalase
CP: Cisplatin
Gapdh: Glyceraldehyde 3-phosphate dehydrogenase
GPx: Glutathione peroxidase
GSH: Glutathione
Gsr: Glutathione reductase
Gss: Glutathione synthase
HE: Hematoxylin and eosin
Il6: Interleukin 6
MDA: Malondialdehyde
m-KRB: modified Krebs−Ringer bicarbonate medium
PB: Phosphate buffer
qPCR: quantitative real-time RT-PCR
RLN: Relaxin
ROS: Reactive oxygen species
RT-PCR: Reverse transcription polymerase chain reaction
RXFP1/Rxfp1: Relaxin family peptide receptor 1
SOD: Superoxide dismutase
TUNEL: Terminal deoxynucleotidyl transferase-mediated deoxyuridine triphosphate-biotin nick end labeling

## References

[1] Dasari S, Tchounwou PB (2014). Cisplatin in cancer therapy: molecular mechanisms of action. Eur J Pharmacol.

[2] Santabarbara G, Maione P, Rossi A, Gridelli C (2016). Pharmacotherapeutic options for treating adverse effects of Cisplatin chemotherapy. Expert Opin Pharmacother.

[3] Quintanilha JCF, de Sousa VM, Visacri MB (2017). Involvement of cytochrome P450 in cisplatin treatment: implications for toxicity. Cancer Chemother Pharmacol.

[4] Cherry SM, Hunt PA, Hassold TJ (2004). Cisplatin disrupts mammalian spermatogenesis, but does not affect recombination or chromosome segregation. Mutat Res.

[5] Martin RH, Ernst S, Rademaker A, Barclay L, Ko E, Summers N (1999). Analysis of sperm chromosome complements before, during, and after chemotherapy. Cancer Genet Cytogenet.

[6] Türk G, Ateşşahin A, Sönmez M, Ceribaşi AO, Yüce A (2008). Improvement of cisplatin-induced injuries to sperm quality, the oxidant-antioxidant system, and the histologic structure of the rat testis by ellagic acid. Fertil Steril.

[7] Howell SJ, Shalet SM (2005). Spermatogenesis after cancer treatment: damage and recovery. J Natl Cancer Inst Monogr.

[8] Sherwood OD, Knobil E, Neill JD (1994). Relaxin. The physiology of reproduction.

[9] Kohsaka T, Sasada H, Watanabe S, Sato E, Bamba K, Sato E, Miyamoto H, Manabe N (2003). Recent advances in research on the hormone relaxin. Animal Frontier Sciences.

[10] Sherwood OD (2004). Relaxin’s physiological roles and other diverse actions. Endocr Rev.

[11] Unemori E (2017). Serelaxin in clinical development: past, present and future. Br J Pharmacol.

[12] Ng HH, Leo CH, Prakoso D, Qin C, Ritchie RH, Parry LJ (2017). Serelaxin treatment reverses vascular dysfunction and left ventricular hypertrophy in a mouse model of type 1 diabetes. Sci Rep.

[13] Leo CH, Jelinic M, Ng HH, Parry LJ, Tare M (2019). Recent developments in relaxin mimetics as therapeutics for cardiovascular diseases. Curr Opin Pharmacol.

[14] Yoshida T, Kumagai H, Suzuki A (2012). Relaxin ameliorates salt-sensitive hypertension and renal fibrosis. Nephrol Dial Transplant.

[15] Yoshida T, Kumagai H, Kohsaka T, Ikegaya N (2013). Relaxin protects against renal ischemia-reperfusion injury. Am J Physiol Renal Physiol.

[16] Yoshida T, Kumagai H, Kohsaka T, Ikegaya N (2014). Protective effects of relaxin against cisplatin-induced nephrotoxicity in rats. Nephron Exp Nephrol.

[17] Bani D, Masini E, Bello MG, Bigazzi M, Sacchi TB (1998). Relaxin protects against myocardial injury caused by ischemia and reperfusion in rat heart. Am J Pathol.

[18] Perna AM, Masini E, Nistri S (2005). Novel drug development opportunity for relaxin in acute myocardial infarction: evidences from a swine model. FASEB J.

[19] Samuel CS, Cendrawan S, Gao XM (2011). Relaxin remodels fibrotic healing following myocardial infarction. Lab Investig.

[20] Anderson MB, Collado-Torres M, Vaupel MR (1986). Absence of relaxin immunostaining in the male reproductive tracts of the rat and mouse. J Histochem Cytochem.

[21] Kato S, Siqin MI (2010). Evidence for expression of relaxin hormone-receptor system in the boar testis. J Endocrinol.

[22] Hsu SY, Nakabayashi K, Nishi S (2002). Activation of orphan receptors by the hormone relaxin. Science.

[23] Kwan TK, Poh CH, Perumal R, Gower DB (1994). Pregnenolone metabolism in testicular homogenates of macaques (Macaca fascicularis): some effects of relaxin and freezing. Biochem Mol Biol Int.

[24] Samuel CS, Tian H, Zhao L, Amento EP (2003). Relaxin is a key mediator of prostate growth and male reproductive tract development. Lab Investig.

[25] Kohsaka T, Takahara H, Sugawara K, Tagami S (1993). Endogenous heterogeneity of relaxin and sequence of the major form in pregnant sow ovaries. Biol Chem Hoppe Seyler.

[26] Minagawa I, Sagata D, Pitia AM (2014). Dynamics of insulin-like factor 3 and its receptor expression in boar testes. J Endocrinol.

[27] Johnsen SG (1970). Testicular biopsy score count – a method for registration of spermatogenesis in human testes: normal values and results in 335 hypogonadal males. Hormones.

[28] Cosentino MJ, Nishida M, Rabinowitz R, Cockett AT (1986). Histopathology of prepubertal rat testes subjected to various durations of spermatic cord torsion. J Androl.

[29] Pfaffl MW, Dorak T (2006). Relative quantification. Real-time PCR.

[30] Richburg JH, Nañez A (2003). Fas- or FasL-deficient mice display an increased sensitivity to nitrobenzene-induced testicular germ cell apoptosis. Toxicol Lett.

[31] Hess HH, Lees MB, Derr JE (1978). A linear Lowry-Folin assay for both water-soluble and sodium dodecyl sulfate-solubilized proteins. Anal Biochem.

[32] Johansson LH, Borg LA (1988). A spectrophotometric method for determination of catalase activity in small tissue samples. Anal Biochem.

[33] Yazdanparast R, Bahramikia S, Ardestani A (2008). Nasturtium officinale reduces oxidative stress and enhances antioxidant capacity in hypercholesterolaemic rats. Chem Biol Interact.

[34] Ellman GL (1959). Tissue sulfhydryl groups. Arch Biochem Biophys.

[35] Moore HD, Akhondi MA (1996). Fertilizing capacity of rat spermatozoa is correlated with decline in straight-line velocity measured by continuous computer-aided sperm analysis: epididymal rat spermatozoa from the proximal cauda have a greater fertilizing capacity in vitro than those from the distal cauda or vas deferens. J Androl.

[36] Toyoda Y, Chang MC (1974). Fertilization of rat eggs in vitro by epididymal spermatozoa and the development of eggs following transfer. J Reprod Fertil.

[37] Sasaki Y, Kohsaka T, Kawarasaki T (2001). Immunoreactive relaxin in seminal plasma of fertile boars and its correlation with sperm motility characteristics determined by computer-assisted digital image analysis. Int J Androl.

[38] Sagata D, Minagawa I, Kohriki H (2015). The insulin-like factor 3 (INSL3)-receptor (RXFP2) network functions as a germ cell survival/anti-apoptotic factor in boar testes. Endocrinology.

[39] Minagawa I, Murata Y, Terada K (2018). Evidence for the role of INSL3 on sperm production in boars by passive immunisation. Andrologia.

[40] Casares C, Ramírez-Camacho R, Trinidad A, Roldán A, Jorge E, García-Berrocal JR (2012). Reactive oxygen species in apoptosis induced by cisplatin: review of physiopathological mechanisms in animal models. Eur Arch Otorhinolaryngol.

[41] Wei SM, Yan ZZ, Zhou J (2007). Beneficial effect of taurine on testicular ischemia-reperfusion injury in rats. Urology.

[42] Zhang X, Lv F, Tang J (2016). Protection from ischemia by preconditioning, postconditioning, and combined treatment in rabbit testicular ischemia reperfusion injury. Arch Biochem Biophys.

[43] Ilbey YO, Ozbek E, Cekmen M, Simsek A, Otunctemur A, Somay A (2009). Protective effect of curcumin in cisplatin-induced oxidative injury in rat testis: mitogen-activated protein kinase and nuclear factor-kappa B signaling pathways. Hum Reprod.

[44] Afsar T, Razak S, Khan MR, Almajwal A (2017). Acacia hydaspica ethyl acetate extract protects against cisplatin-induced DNA damage, oxidative stress and testicular injuries in adult male rats. BMC Cancer.

[45] Ateşşahin A, Karahan I, Türk G, Gür S, Yilmaz S, Ceribaşi AO (2006). Protective role of lycopene on cisplatin-induced changes in sperm characteristics, testicular damage and oxidative stress in rats. Reprod Toxicol.

[46] Saral S, Ozcelik E, Cetin A (2016). Protective role of Diospyros lotus on cisplatin-induced changes in sperm characteristics, testicular damage and oxidative stress in rats. Andrologia.

[47] Amin A, Hamza AA (2006). Effects of Roselle and Ginger on cisplatin-induced reproductive toxicity in rats. Asian J Androl.

[48] Silici S, Ekmekcioglu O, Eraslan G, Demirtas A (2009). Antioxidative effect of royal jelly in cisplatin-induced testes damage. Urology.

[49] Aksu EH, Kandemir FM, Özkaraca M, Ömür AD, Küçükler S, Çomaklı S (2017). Rutin ameliorates cisplatin-induced reproductive damage via suppression of oxidative stress and apoptosis in adult male rats. Andrologia.

[50] Nistri S, Cinci L, Perna AM, Masini E, Mastroianni R, Bani D (2008). Relaxin induces mast cell inhibition and reduces ventricular arrhythmias in a swine model of acute myocardial infarction. Pharmacol Res.

[51] Sasser JM, Cunningham MW, Baylis C (2014). Serelaxin reduces oxidative stress and asymmetric dimethylarginine in angiotensin II-induced hypertension. Am J Physiol Renal Physiol.

[52] Kamat AA, Feng S, Bogatcheva NV, Truong A, Bishop CE, Agoulnik AI (2004). Genetic targeting of relaxin and insulin-like factor 3 receptors in mice. Endocrinology.

[53] García MM, Acquier A, Suarez G (2012). Cisplatin inhibits testosterone synthesis by a mechanism that includes the action of reactive oxygen species (ROS) at the level of P450scc. Chem Biol Interact.

[54] Smith LB, Walker WH (2014). The regulation of spermatogenesis by androgens. Semin Cell Dev Biol.

[55] Lenzi A, Gandini L, Maresca V, Rago R, Sgrò P, Dondero F, Picardo M (2000). Fatty acid composition of spermatozoa and immature germ cells. Mol Hum Reprod.

[56] Gavella M, Lipovac V (2013). Protective effects of exogenous gangliosides on ROS-induced changes in human spermatozoa. Asian J Androl.

[57] El-Shafaei A, Abdelmaksoud R, Elshorbagy A, Zahran N, Elabd R (2018). Protective effect of melatonin versus montelukast in cisplatin-induced seminiferous tubule damage in rats. Andrologia.

