# Supplementary material for: Efficacy of relaxin for cisplatin-induced testicular dysfunction and epididymal spermatotoxicity
Source: Basic Clin Androl. 2020 Mar 9;30:3. doi: 10.1186/s12610-020-0101-y (PMC7061478; doi:10.1186/s12610-020-0101-y)
Supplement: Supplementary file 1 [file 12610_2020_101_MOESM1_ESM.docx]

Supplemental Table 1. List of primers employed for quantitative real-time RT-PCR in rat target genes.

Gene	Purpose	Forward primer (5' → 3')	Reverse primer (3' → 5')	Annealing (°C)	Product (bp)	GenBank accession no.
Rxfp1	qPCR	ggaaatgcacaggatgtcagt	ggaagtttctgcctcaatgg	60	222	NM_201417
Il6	qPCR	attgtatgaacagcgatgatgcac	ccaggtagaaacggaactccaga	60	150	NM_012589
Gapdh	qPCR	tcaagaaggtggtgaagcag	tgggagttgctgttgaagtc	60	100	NM_017008
